# A Randomised, Parallel-Group Study to Compare the Efficacy of 3% Phenothrin-Containing Jigger Lotion Versus Potassium Permanganate for Treatment of Tungiasis in Vihiga County, Kenya

**DOI:** 10.3390/tropicalmed11020047

**Published:** 2026-02-09

**Authors:** Kana Suzuki, Asiko Ongaya, Evans Amukoye, Yasuhiko Kamiya

**Affiliations:** 1 Institute of Tropical Medicine, Nagasaki University, Nagasaki 852-8523, Japan; suzuki.kan.za@yokohama-cu.ac.jp; 2 Nursing Course, School of Medicine, Yokohama City University, Yokohama 236-0004, Japan; 3 Kenya Medical Research Institute, Nairobi 54840 00200, Kenya; 4 School of Tropical Medicine and Global Health, Nagasaki University, Nagasaki 852-8523, Japan

**Keywords:** tungiasis, randomised controlled trial, topical treatment, 3% phenothrin lotion, potassium permanganate, resource-limited setting, Kenya

## Abstract

Tungiasis, caused by the sand flea *Tunga penetrans*, results in itching and pain. Effective treatments, such as dimeticones, are often unaffordable. A 3% phenothrin lotion has shown safety and efficacy in Kenyan trials. This study compared the cure rate and safety of 3% phenothrin lotion (as the intervention) and 0.05% potassium permanganate (KMnO_4_; as the standard-care comparator) over 14 days. This parallel-group, three-arm, non-blinded, randomised comparative trial was conducted in Vihiga County, Kenya. Participants aged ≥2 years with ≥1 viable flea on each foot were allocated (2:1:1) to KMnO_4_, single-dose 3% phenothrin, or two-dose 3% phenothrin groups. Overall, 415 fleas from 79 participants were followed up to day 14 (KMnO_4_, 213; single-dose, 129; two-dose, 73). On days 4 and 7, the single-dose phenothrin showed significantly higher cure rates (11.6% and 21.7%) than KMnO_4_ (0.9% and 11.7%) (*p* < 0.001 and *p* = 0.013). The differences diminished by days 10 and 14 because of spontaneous flea death. The cure rate of the two-dose group on day-7 (8.2%) was lower than that of the single-dose group. Single-dose 3% phenothrin improved early cure rates compared to KMnO_4_, but not by days 10–14; two-dose phenothrin showed no benefit compared with single dose from day 7 onwards.

## 1. Introduction

Tungiasis is a neglected tropical skin disease caused by the sand flea *Tunga penetrans* and, less commonly, *Tunga trimamillata* [1,2]. *T. penetrans*, the most widely distributed *Tunga* species and the one with the broadest host range, is endemic in sub-Saharan Africa, South America, and some islands of the Caribbean. In non-endemic Western countries, tungiasis is mainly recognised as an imported condition [3]. *T. trimamillata* has reported been to be endemic in Peru, Ecuador, and Brazil [2]. Tungiasis has been targeted for control by 2030 in the World Health Organisation’s Neglected Tropical Diseases Road Map, alongside scabies and other ectoparasitoses [4]. Sand fleas burrow into the skin and induce an inflammatory response, leading to itching and pain and, in severe cases, secondary infection and mobility limitations, with substantial impacts on quality of life [1]. Based on a prior review article [5], male sex, earthen floors, and poor knowledge were identified as significant risk factors for tungiasis in Africa.

Currently, treatment options for tungiasis remain limited. A recent systematic review of randomised controlled trials assessing tungiasis interventions [6] reported that a coconut oil-based lotion for prevention [7] and dimeticones (NYDA^®^) for treatment showed the greatest potential for managing tungiasis [8,9]. A study in Kenya reported that dimeticones achieved an approximately 78% cure rate in the treatment of *T. penetrans* infection [8]. In addition, a study in Uganda reported that dimeticones resulted in the death of >95% of fleas [9]. However, the high price of dimeticones makes them unaffordable for many people in Kenya, particularly in low-income households. Meanwhile, locally available treatments have shown lower or inconclusive cure rates [6,7]. The extraction of embedded fleas using non-sterile sharp instruments remains common in endemic areas [10]. Households with low-income are disproportionately affected by tungiasis but often lack access to safe and effective treatment.

A 3% phenothrin lotion, containing phenothrin (a synthetic pyrethroid) as the active ingredient, has recently been developed for the treatment of tungiasis. Phenothrin causes nerve depolarisation and hyperexcitation by acting on voltage-gated sodium channels in the insect nervous system, followed by muscle paralysis and death. In Japan, phenothrin lotion is covered by the national health insurance system and is therefore commonly used in routine clinical practice for the treatment of scabies [11] and headlice [12].

The safety of 3% phenothrin lotion, also known as 5% S-1555 Lotion, was assessed in a phase 1 study conducted in Kiambu County, Kenya; no adverse effects on skin condition or liver function were reported [13]. A phase 2 trial [14], conducted in Busia, Nyeri, and Siaya counties in Kenya in a small cohort, found that 3% phenothrin lotion was more efficacious than 0.05% potassium permanganate (KMnO_4_) solution, the standard treatment currently recommended in Kenya [15]. Based on its favourable safety profile and promising preliminary efficacy, 3% phenothrin lotion was selected for further evaluation in the Kenyan setting. In Kenya, a 0.05% KMnO_4_ solution is included in the national clinical guidelines [15] and remains the most commonly used topical treatment for tungiasis. This concentration has been widely used in previous studies [7,8] and clinical practice, with evidence supporting its efficacy, and was therefore selected as the comparator in this study.

However, comparative evidence from adequately powered clinical trials evaluating 3% phenothrin lotion against KMnO_4_ under field conditions in Kenya remains limited. This study aimed to evaluate the cure rate of 3% phenothrin lotion against *T. penetrans* compared with that of KMnO_4_ in Kenya. The findings are expected to contribute to the development of sustainable, effective, and safe topical treatments in Kenya.

The general objective was to evaluate the cure rates of 3% phenothrin lotion and KMnO_4_ for the topical treatment of tungiasis in western Kenya. The specific objectives were to compare cure rates between 3% phenothrin lotion and KMnO_4_ on days 4, 7, 10, and 14 post-treatment and to assess safety by evaluating the incidence of adverse reactions and events.

## 2. Materials and Methods

### 2.1. Study Design

**Figure 1:**
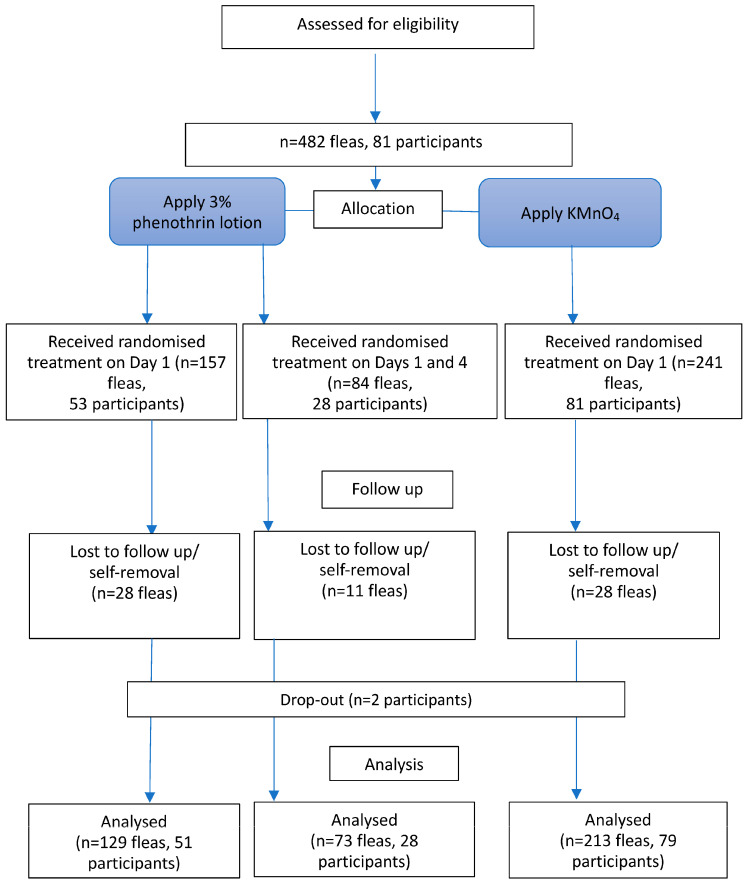
Flowchart of the trial. KMnO_4_, potassium permanganate.

This study was a parallel-group, three-arm, superiority, non-blinded, randomised comparative trial with a 2:1:1 allocation ratio (KMnO_4_:single-dose 3% phenothrin lotion:two-dose 3% phenothrin lotion). The trial process is illustrated in the flowchart (Figure 1). Blinding of participants was not feasible because of differences in product appearance and application, particularly because the KMnO_4_ solution stained the skin for several days. A placebo control group was not included, primarily because of ethical concerns about withholding treatment from individuals with painful and symptomatic tungiasis.

For tungiasis, some interventions, including KMnO_4_, are already in use despite partial effectiveness. In addition, selection of an appropriate placebo is technically challenging. Substances such as oils, lipids, and other emulsifiers used as lotion bases may have plausible effects (e.g., suffocating embedded fleas) and therefore would not serve as truly inert controls. Such effects could confound efficacy assessment. Therefore, KMnO_4_, which is widely used in the region despite partial effectiveness, was selected as the comparator.

### 2.2. Study Setting

This study was conducted in the catchment area of the Emusire Sub-County Hospital and Ipali Health Centre, Emuhaya Sub-County, Vihiga County, western Kenya, where tungiasis is highly prevalent. A previous study reported a 21.5% prevalence in parts of Vihiga County [16]. The site was selected based on a preliminary Ministry of Health survey that identified it as a high-burden area. Recruitment began on 13 March 2024 and data collection was concluded on 16 April 2024 during the rainy season.

### 2.3. Study Participants

Participants were residents of the study area with *T. penetrans* infection who provided consent (or assent, as applicable).

Eligibility criteria included individuals aged ≥2 years with at least one embedded viable flea on each foot, classified as Fortaleza stage II or III (Table 1) [17]. Flea viability was defined by the presence of at least one of the following signs: abdominal cone movement, abdominal pulsations/contractions, expulsion of eggs, faecal thread excretion, or faecal liquid excretion. These signs were assessed by observing the embedded flea for 10 min using a handheld digital video microscope with 5-megapixel optical resolution.

Exclusion criteria included severe tungiasis signs or symptoms, such as marked inflammation; ≥20 fleas; abscess; ascending lymphangitis; or lymphedema on either foot, as well as any disability that prevented active participation in the study. Individuals meeting these criteria were referred to a sub-county or county hospital for further clinical management. Participants with known allergy to any of the study treatments were also excluded. HIV status was not actively screened; however, individuals living with HIV were eligible unless they presented with a severe general condition.

### 2.4. Sample Size

The primary outcome was the cure rate, defined as the proportion of non-viable fleas following treatment with 3% phenothrin lotion or KMnO_4_. Previous studies reported flea mortality of 39% with KMnO_4_ [8] and 67% with 3% phenothrin lotion [14], corresponding to an absolute difference of 28%. A 2:1:1 allocation ratio (KMnO_4_:single-dose 3% phenothrin lotion:two-dose 3% phenothrin lotion) was used to ensure sufficient precision for analysis of the primary outcome.

The original plan was to compare a single dose of 3% phenothrin lotion with KMnO_4_ with respect to efficacy in killing embedded fleas. To allow additional assessment of a two-dose regimen, a subset of participants assigned to phenothrin received a second dose while maintaining the minimum number of lesions required to evaluate the primary outcome. Due to the three-group comparison, a closed testing procedure was used.

The sample size was calculated using Easy R (EZR), a modified version of R Commander that includes statistical functions frequently used in biostatistics [18]. Assuming a cure rate of 39% for KMnO_4_ (P_2_ = 0.39) and 67% for 3% phenothrin lotion (P_2_ = 0.67), with a two-sided α of 0.05 and power of 0.90, the minimum required sample size was estimated as 72 lesions per group. Accounting for the design effect arising from the assessment of one to five lesions per foot per participant and allowing for 10% loss to follow-up, a total of 360 lesions (180:90:90 per group) were required.

### 2.5. Sampling and Recruitment

#### 2.5.1. Sampling of Health Facilities

Endemic schools were mapped in collaboration with the Ministry of Health. Community units (CUs) were ranked according to the reported number of tungiasis cases, with those reporting the highest number of cases selected. The nearest and largest health facilities within these CUs were designated as study sites.

#### 2.5.2. Recruitment of Participants

Snowball sampling was used via eligible participants and community health promoters (CHPs; known as community health volunteers in Kenya), in accordance with the predefined inclusion and exclusion criteria. Potential participants were contacted by a local co-investigator and field assistants after a clear explanation of the study objectives and procedures. Trained CHPs and Community Health Assistants working near the selected facilities mobilised their communities and invited residents to attend treatment campaigns at the facilities. Participants were enrolled on a first-come, first-served basis, irrespective of age. Eligible participants received free treatment, transport reimbursement of 200 KSh (approximately USD 1.5), and a pair of sandals if they were barefoot.

### 2.6. Randomisation

Lesions caused by embedded viable fleas were identified before randomisation to reduce selection bias. Each participant’s foot was then randomly assigned to either 3% phenothrin lotion (single-dose or two-dose) or KMnO_4_ using a sealed-envelope allocation method.

### 2.7. Interventions

Trained nurses or clinical officers administered the interventions. Before treatment, participants’ feet were thoroughly washed with soap and water and dried with a clean towel. The 3% phenothrin lotion was applied to both feet up to the ankle. The pump-type product has a capacity of 480 mL and dispenses approximately 1 g per push (equivalent to two fingertip units [FTUs]). The following amounts were applied per foot according to participant age: 2 FTUs (1 push) for ages 2–5 years, 4 FTUs (2 pushes) for ages 6–10 years, and 6 FTUs (3 pushes) for ages ≥11 years. Treatment was administered once in the single-dose group and repeated on day 4 in the two-dose group.

Participants assigned to the KMnO_4_ group soaked their feet up to the ankles in a bucket containing 5.0 L of 0.05% KMnO_4_ solution for 15 min. After the feet were dried, petroleum jelly (Vaseline; Unilever, London, UK) was applied to prevent skin desiccation, and all identified lesions were treated.

### 2.8. Outcomes

The primary outcome was the cure rate, defined as the proportion of embedded fleas assessed as non-viable (dead) following treatment with 3% phenothrin lotion or KMnO_4_. Embedded fleas were considered dead if no signs of viability (e.g., egg expulsion; excretion of faecal threads or liquid; or visible pulsations or contractions) were observed during a 10 min observation period.

The secondary outcome was the occurrence of adverse effects and reactions during the 14-day monitoring period, compared between treatment groups. Monitored symptoms included itching, pain, tenderness, and sleep disturbance. Monitored signs included erythema, warmth, oedema, desquamation, ulcers, fissures, suppuration, abscesses, and pustules. The severity of itching and pain was rated on a 4-point scale: none, only a little, quite a lot, and very much.

### 2.9. Data Collection

**Figure 2:**
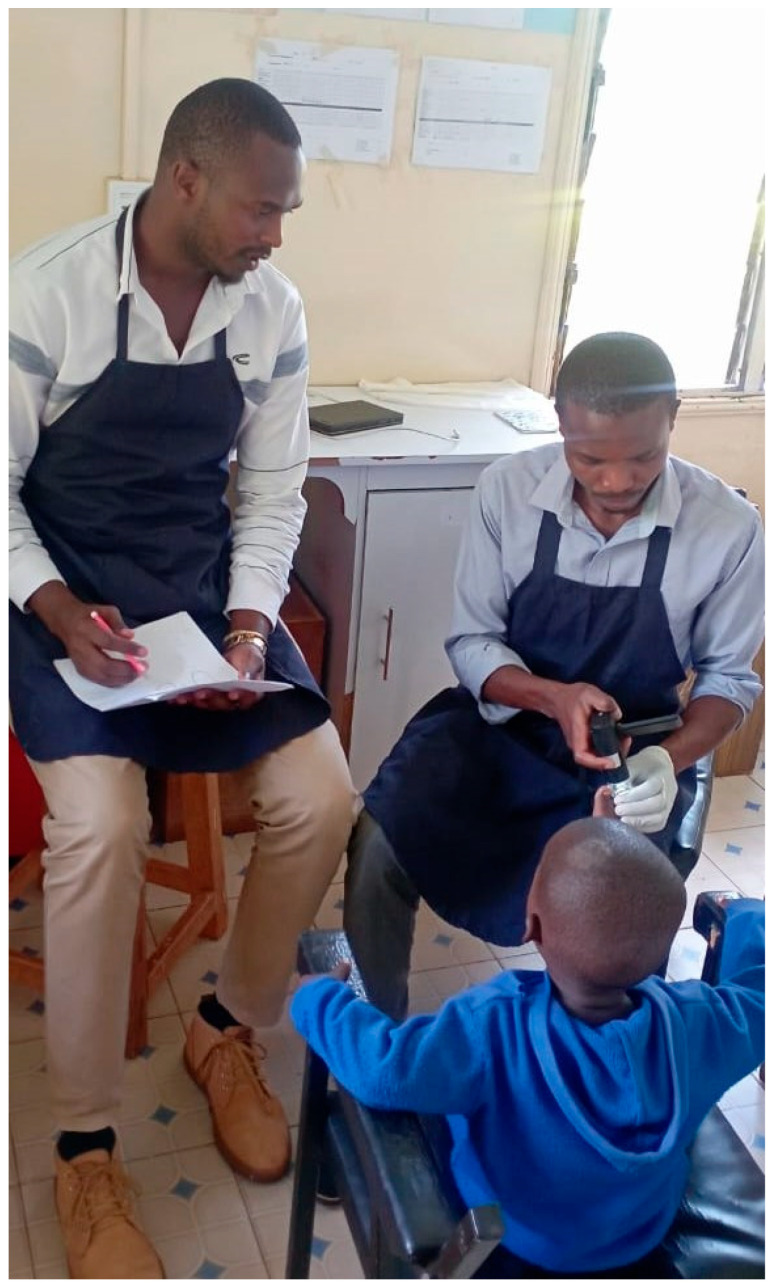
Observation of flea viability by a laboratory technician, with a nurse recording the findings.

Trained research nurses or clinical officers and laboratory technicians collected the data (Figure 2). The viability of embedded fleas in each participant was assessed using a handheld microscope. Baseline demographic data and clinical and behavioural characteristics were obtained through interviews using a structured questionnaire translated into the local language. Additional measurements included body weight, height, heart rate, respiratory rate, and blood pressure. Lesions were inspected, monitored, and recorded using case observation and recording forms.

### 2.10. Data Management

All data were recorded electronically on password-protected smartphones using REDCap Version 12.5.9 (Kenya Medical Research Institute, Nairobi, Kenya). When not in use, the smartphones were stored in a locked cabinet in a locked room. No participant data remained on the smartphone hard drive once data entry was confirmed. Encrypted data were then uploaded from the smartphones to a secure backup server. Data quality and confidentiality were continuously monitored by the principal investigator and co-investigators.

### 2.11. Monitoring

Participants were monitored on days 1, 4, 7, 10, and 14 over a 14-day period after enrolment. They were instructed not to remove fleas or attempt self-treatment during the study period. In addition to investigator monitoring of data quality and safety, an independent monitor oversaw the study to confirm appropriate implementation and data reliability.

### 2.12. Data Analyses

The per-protocol set was used to analyse the primary outcome. Only fleas from participants with good treatment adherence and complete 14-day follow-up were included. The unit of analysis was the individual flea, and the cure rate was defined as the proportion of fleas classified as dead on day 14. Fleas were excluded if their viability status (alive or dead) could not be confirmed at follow-up (e.g., if they had been removed or were no longer visible). These were treated as missing data at the flea level and excluded from the final analysis. A chi-square test was used to assess differences in cure rates between the 3% phenothrin lotion and KMnO_4_ groups. Absolute differences in cure rates were calculated to estimate between-group differences in efficacy. The log-rank test was used to compare time to cure (flea death) among the three groups, with follow-up censored at day 14 after the initial treatment. Relative risks with corresponding 95% confidence intervals (CIs) were calculated to compare the incidence of adverse effects between treatment groups. For all tests, two-sided *p* values were used, with α = 0.05 as the level of significance. Statistical analyses were performed using EZR software (version 1.54) [18].

## 3. Results

### 3.1. Recruitment and Follow-Up

In total, 81 eligible individuals infested with 482 embedded fleas at stages 2 or 3 were enrolled. Fleas on either the left or right foot were assigned to the KMnO_4_ or 3% phenothrin lotion treatment groups. Among them, 84 fleas from 28 participants were assigned to receive an additional dose of 3% phenothrin lotion on the 4th day. After the assignment, two participants were lost to follow-up. Additionally, 28 fleas were lost in the KMnO_4_ treatment group, whereas 28 and 11 fleas were lost in the single-dose and two-dose 3% phenothrin lotion groups, respectively, presumably removed by the participants. Subsequently, 415 fleas from 79 participants were followed up until the death of the fleas or on the 14th day. All participants received KMnO_4_ and a 3% phenothrin lotion (at least one dose) on either their left or right foot. Of these, 213 fleas in 79 participants were treated with KMnO_4_, 129 fleas in 51 participants received a single dose of 3% phenothrin lotion, and the remaining 73 fleas in 28 participants received a second dose of 3% phenothrin lotion on the 4th day. The process described above is illustrated in the flowchart in Figure 1. Consequently, the number of fleas and participants in the two-dose group was smaller than initially planned, which may have limited the statistical power to detect differences for this group.

### 3.2. Profile of Study Participants

The ages of the 79 participants ranged from 2 to 72 years (median age: 11 years); 62 participants were aged <18 years and 3 were aged ≥60 years.

Approximately 40% of the participants were younger than 10 years, and more than three-quarters of the participants were aged <20 years, as summarised in Table 2. This study included 48 men and 31 women. Ten participants had dermatological signs other than tungiasis lesions; however, these lesions were not severe enough to require medical treatment.

Approximately half of the participants wore shoes regularly. Around the third quarter of the study, the participants washed their feet once daily. Over 90% of the participants had experienced self-removal of embedded sand fleas. Only eight and six participants owned pigs and chickens, respectively. The distributions of other possible risk factors are presented in Table 2 and Table 3.

### 3.3. Primary Outcome

**Figure 3:**
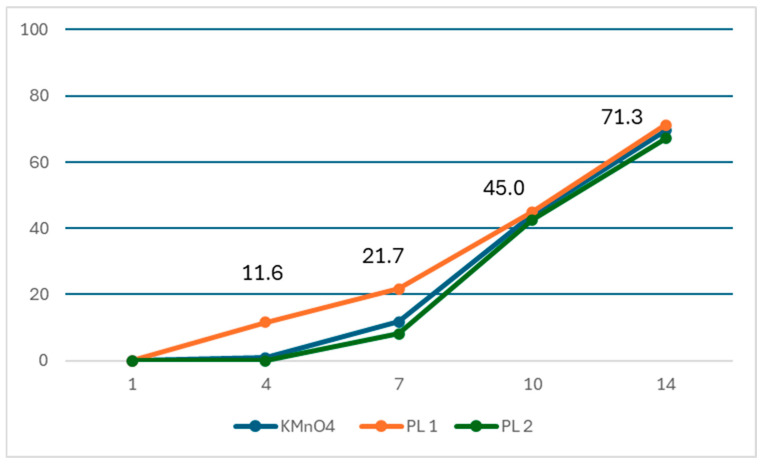
Proportion of dead sand fleas (cure rate) over 14 days in KMnO_4_, single-dose 3% phenothrin lotion (PL1), and two-dose 3% phenothrin lotion (PL2) treatment groups. KMnO_4_, potassium permanganate.

On the 4th day, 15 (11.6%) and 2 fleas (0.9%) were observed to be dead in the single-dose 3% phenothrin lotion and KMnO_4_ treatment groups, respectively, as shown in Table 4 and Figure 3. The absolute cure rate difference was 10.7% (95% CI: 5.0–16.4) (Table 5). The odds ratio for the cure rate was 13.88 (95% CI: 3.12–61.77) for 3% phenothrin lotion compared with KMnO_4_ (Supplementary Table S1). On the 7th day, 28 fleas (21.7%) and 25 fleas (11.7%) were observed to be dead in the single-dose 3% phenothrin lotion and KMnO_4_ treatment groups, respectively. The absolute cure rate difference between the two groups was 10.0% (95% CI: 1.6–18.3), which was statistically significant (*p* = 0.013). The odds ratio for the cure rate was 2.08 (95% CI: 1.15–3.76) for 3% phenothrin lotion compared with KMnO_4_ (Supplementary Table S1). The cure rate for the two-dose 3% phenothrin lotion on the 7th day was 8.2%. The absolute cure rate difference between the single-dose and two-dose 3% phenothrin lotion was 13.5% (95% CI: 3.9–23.5), which was statistically significant (*p* = 0.005). On the 10th day, the cure rate for KMnO_4_ was 44.1%, whereas the cure rates for the single-dose and two-dose 3% phenothrin lotion were 45.0% and 42.5%, respectively. However, these differences were not statistically significant. The cure rates on the 14th day with KMnO_4_, single-dose 3% phenothrin lotion, and two-dose 3% phenothrin lotion were 69.5%, 71.3%, and 67.1%, respectively, which were not significantly different (Figure 3).

### 3.4. Secondary Outcomes

No serious adverse events were observed. A significant number of participants in both groups complained of varying degrees of itching or pain. However, erythema, warmth, oedema, desquamation, ulcers, fissures, suppurations, ulcers, abscesses, and pustules around the flea lesions were not observed.

Itching and pain complaints by the study participants were rated on four scales: none, only a little, quite a lot, and very much; however, no participants reported ‘very much’ for itching or pain. Severity was categorised into two levels for the analysis: non-severe (none and only a little) and severe (quite a lot and very much). The results of the analysis regarding the incidence of itching and pain complaints are summarised in Table 6 and Table 7. At baseline, 16.5% and 3.8% of participants reported severe itching and severe pain, respectively, in the KMnO_4_ group.

On the 4th day, eight (10.1%) participants in the KMnO_4_ group and seven (13.7%) in the single-dose 3% phenothrin lotion group reported severe itching; this difference was not statistically significant. Furthermore, five (6.3%) participants in the KMnO_4_ group and four (7.8%) in the single-dose 3% phenothrin lotion group complained of severe pain, which was not significantly different. Notably, no participants in the two-dose 3% phenothrin lotion treatment group complained of severe itching or pain; however, 24 (86%) and 16 (57%) of the 29 participants experienced mild itching and pain, respectively, on the 4th day (Supplementary Tables S2 and S3). Fisher’s exact test revealed no statistically significant differences in severe itching and pain among the KMnO_4_, single-dose 3% phenothrin lotion, and two-dose 3% phenothrin lotion groups (itching, *p* = 0.1077; pain, *p* = 0.3231).

The rate of severe itching and pain on the 7th day was 11.4% in the KMnO_4_ treatment group compared with 3.9% and 3.6% in the single-dose and two-dose 3% phenothrin lotion treatment groups, respectively. The relative risk ratios for itching and pain were 0.34 and 0.31 for the single-dose and two-dose 3% phenothrin lotion groups, respectively, compared to that for KMnO_4_. However, this difference was not statistically significant (*p* = 0.135 and 0.221 for itching and pain, respectively; Supplementary Tables S2 and S3). The differences in the rates of itching and pain between the KMnO_4_ and 3% phenothrin lotion treatment groups on days 10th and 14th days were smaller than on the 7th day and were not statistically significant.

## 4. Discussion

Our study findings demonstrated that a single-dose 3% phenothrin lotion differed significantly from KMnO_4_ on Days 4 and 7, whereas two doses of phenothrin showed no difference. On day 7, there was a significant difference, with the single dose being more effective than the two doses. This unexpected finding should be interpreted with caution and primarily regarded as hypothesis-generating. This two-dose result may be due to chance or the small sample size. It is possible that repeated applications, specifically two doses, may have reduced insecticidal efficacy, potentially related to local accumulation of phenothrin in the skin [19] and altered transcutaneous penetration into embedded fleas; however, this explanation remains speculative. Additionally, the observed difference may be attributable to random variations, particularly given the limited sample size. Although the results were statistically significant, the possibility of a chance finding cannot be entirely ruled out.

Phenothrin, the active ingredient in 3% phenothrin lotion, is commonly utilised to kill household insects such as lice [11]. It is a fast-acting insecticide that works through contact and ingestion, is rapidly metabolised and excreted by mammals, and has low mammalian toxicity [20].

In this study, 3% phenothrin lotion and KMnO_4_ took 3–7 days longer to kill embedded fleas compared to the time recorded in previous studies on tungiasis treatment using phenothrin [13,14]. Specifically, a 3% phenothrin lotion killed 11.6% of embedded fleas by the 4th day, whereas a phase 2 trial using a 5% phenothrin lotion reported a 68% flea mortality rate at the same time point [13,14]. This difference in the onset of the insecticidal effect may be attributable not only to the concentration difference (5% vs. 3%) but also to potential geographical variation in flea susceptibility or resistance to insecticides. Such variability may result from regional genetic diversity among *T. penetrans* populations. Similar differences in susceptibility have been documented in other arthropod species, which could influence the effectiveness of phenothrin-based treatments across different settings. Another possible reason is that the thick and rough skin of the study participants, half of whom did not wear shoes regularly, may have prevented smooth and rapid penetration of the 3% phenothrin lotion and KMnO_4_ into the embedded fleas targeted by the treatment [9]. Hence, the slow progress in the cure rate observed in this study may reflect a thicker and rougher corneal layer of the skin affected by tungiasis. Further entomological and molecular studies are needed to investigate the potential resistance patterns in flea populations across regions. Furthermore, the results of the present study showed a relatively lower cure rate than the 7-day cure rates obtained in previous clinical studies. Specifically, the cure rates for the 3% phenothrin lotion (single dose) and KMnO_4_ were 21.7% and 11.7%, respectively. In contrast, previously reported cure rates include NYDA at 78%, 87%, and 97% [8,9,21], sodium carbonate at 64% [21], and a combination of coconut oil and neem oil at 30% [7]. However, compared with the cure rates recorded in previous studies using KMnO_4_, which reported cure rates of 39% and 40% [7,8], the low cure rates observed in this study may be influenced by the specific lifestyle and living conditions of the study participants. The fact that KMnO_4_ also showed reduced efficacy in this setting suggests that a 3% phenothrin lotion may yield better outcomes in different populations or environments. Another noteworthy factor was the use of Vaseline. The amount applied was not standardised, and its consistency can vary depending on environmental temperature and body heat, affecting its melting and spread. Once melted, Vaseline may contribute to treatment by suffocating fleas. However, no studies have clearly defined the appropriate quantity or assessed its efficacy, which could account for the discrepancies between our results and those of other studies involving KMnO_4_.

Nevertheless, the ability of the 3% phenothrin lotion to kill embedded fleas earlier than KMnO_4_ could result in a quicker resolution of acute inflammation. This may help alleviate acute symptoms such as pain and itching. Additionally, early flea death can prevent the release of eggs, thereby reducing the risk of reinfection.

Thus, the 3% phenothrin lotion has advantages over KMnO_4_, a locally available treatment recommended by the national guidelines in Kenya, which is slower in killing fleas and can cause skin irritation. Sodium carbonate is another treatment available in Kenya that can kill fleas faster; however, this alkaline agent may cause skin irritation [21]. A mixture of two dimeticones with different viscosities and volatilities, designed to occlude the rear abdominal cone of the embedded flea [8,9], is listed in the treatment section of the National Policy Guidelines for Prevention and Control of Jigger Infestations 2014 in Kenya [15]. However, dimeticones are expensive and currently unavailable in tungiasis-endemic areas, including Kenya.

In addition, we observed a diminishing difference in flea mortality rates between the 3% phenothrin lotion and KMnO_4_ over time on days 10 and 14. This was partly a result of spontaneous death, irrespective of treatment. As acute local inflammation is causally associated with the presence of viable embedded fleas, acute symptoms and signs rapidly regress once the fleas die and disappear within a short period. Dead fleas are eliminated by natural skin repair mechanisms. However, nail deformation and hypertrophy of the nail rim may persist as chronic complications in severe cases or in patients with poor nutritional or immunological conditions, even after the death of fleas. Hence, long-term follow-up and care are crucial in such cases. Nevertheless, the early superiority of single-dose phenothrin suggests its potential value as an early symptomatic treatment. However, its price has not yet been established in the Kenyan market, and accessibility remains uncertain.

This is an important consideration for ensuring patient comfort and treatment compliance. No statistically significant differences were found in adverse effects between the treatment groups. Additionally, the safety of the 3% phenothrin lotion has been confirmed in a phase I study, including a biochemical examination showing no elevation in aspartate aminotransferase and alanine aminotransferase, indicating the absence of adverse effects on liver function [13]. These safety findings support the tolerability of 3% phenothrin lotion.

### Limitations

This study had some limitations. The loss of enrolled fleas due to self-removal by the participants during the follow-up period might have influenced the cure rate results. Additionally, we did not assess the abnormal development of fleas, such as the unnaturally fast ageing process followed by early termination of egg expulsion over the observation period. Monitoring not only death but also abnormal development in fleas could have provided a more detailed understanding of the effects of treatment on symptoms, complications, and transmission. The participants were allocated in a 2:1:1 ratio to ensure sufficient power for the primary outcome. However, a procedural error during allocation resulted in an imbalance in group sizes, with 213, 129, and 73 fleas in the KMnO_4_, single-dose, and two-dose 3% phenothrin groups, respectively. This deviation from the planned allocation reduced the statistical power of the two-dose group. Consequently, comparisons involving this group, including the similarity observed between the KMnO_4_ and two-dose phenothrin groups, should be interpreted with caution. The use of snowball sampling may have introduced selection bias, although efforts such as foot-level randomisation were made to minimise it. In addition, fleas rather than individuals were used as the unit of analysis, although multiple fleas were clustered within the same participant. This may have violated the assumption of independence and could have led to an underestimation of variance, and potentially inflated the type I error rate. While foot-level randomisation was employed to mitigate within-participant correlation, residual clustering effects cannot be fully excluded. Therefore, the results, particularly those involving smaller groups, should be interpreted with caution. It is not possible to draw definitive conclusions regarding whether a single application is sufficient or whether a double dose offers additional benefits. Interestingly, the cure rate on day 7 in the two-dose 3% phenothrin lotion group was significantly lower than that in the single-dose group. Although the reason remains unclear, this may be related to the smaller number of participants recruited in the two-dose group, as noted above. Moreover, the inability to determine the exact cause of flea death on the 10th and 14th days complicates the interpretation of long-term efficacy. Furthermore, in the absence of blinding, this study relied on self-reported measures of itching and pain, which are inherently subjective and prevent objective assessment of symptom location or cause, such as whether they were because of the treatments, fleas’ development, or inflammation. In particular, the visible skin discoloration caused by KMnO_4_ may have influenced the participants’ perception and reporting of symptoms, potentially leading to the overreporting of itching or pain in this group. Although future studies should consider the use of infrared imaging or more detailed symptom tracking (e.g., onset and location of pain or itching), definitive attribution would likely remain difficult. Another limitation is the absence of a placebo arm, which limits our ability to fully attribute the observed effects to the treatment itself.

## 5. Conclusions

A 3% phenothrin lotion offers faster symptom relief and early flea kill compared to KMnO_4_ and may be a useful treatment option in settings where rapid symptom control is a priority. Further research involving a larger study population, more frequent observations, and a broader geographical scope is necessary to verify these findings and better understand the long-term benefits and any potential adverse effects.

## Figures and Tables

**Table 1:** Stages of lesions according to the Fortaleza classification.

Fortaleza Stage	Characteristics	Period
Stage I	Active penetration of the sand flea into the skin	3–7 h
Stage II	Early lesion: Brownish/black dot with a diameter of 1–2 mm surrounded or not by erythema (with or without local pain and itching)	1–2 days
Stage IIIa	Mature egg-producing flea: Circular yellow-white watch glass-like patch with a diameter of 3–10 mm and with a central black dot	2–3 days
Stage IIIb	Viability signs continue to be clear, intermittent egg expulsion, and rim forms around the abdominal cone with a concave surface. Lasts for 2 weeks	2 weeks
Stage IVa	Flea shrinks, involution of the lesion: Brownish-black crust with or without surrounding necrosis	2 weeks
Stage IVb	Viability signs completely absent	1–2 weeks
Stage V	Classic circular scar on the skin	1–2 weeks

**Table 2:** Characteristics of the study participants: Individual risk factors and practices.

	Characteristics	*n* = 79	No. (%) Unless Specified	Details
	Age		Median: 11	<18 years: 62, 18–60 years: 14, >60 years: 3
	Sex	Male	48 (60.8)	
		Female	31 (39.2)	
Individual risk factors	Condition of uniform/other clothes [observation]	Torn/rags	14 (17.7)	
		Moderate	48 (60.8)	
		Smart	16 (20.3)	
	Condition of the patient’s cleanness [observation]	Not clean	38 (48.1)	
		Look bathed and moisturised	41 (51.9)	
	Shoes wearing [observation]	None	31 (39.2)	
		Open slippers	20 (25.3)	
		Closed shoes	25 (31.6)	
		Closed shoes and socks	3 (3.8)	
	Other skin diseases [observation]	Don’t have	69 (87.3)	
		They have	10 (12.7)	
	Regular shoes wearing	Not regularly	39 (49.4)	
		Regularly	40 (50.6)	
	Constant shoes wearing at home or school	Not constantly	38 (48.1)	
		Constantly	40 (50.6)	
	Using soap while washing their feet	No	6 (7.6)	
		Sometimes	69 (87.3)	
		Always	4 (5.1)	
	Frequency of washing their feet	Twice a day	11 (13.9)	
		Once a day	58 (73.4)	
		Less often	10 (12.7)	
	Walking time from home to office or school (min)		mean 33.05	
Practices	Removing jiggers by the patients/parents	Never	4 (5.1)	Thorn: 6; needle: 70; blade: 14
		sometimes	52 (65.8)	
		Regularly	22 (27.8)	
	Other treatments by the patients/parents	No	51 (64.6)	Sprinkle water: 6; spray insecticide: 7; spread fire ash: 2; smear mud: 2; apply body oil/keep individual/environment clean: 11
		Yes	28 (35.4)	
	Practice of the patients/parents to kill jiggers in house	No	14 (17.7)	Sprinkle water: 46; spray insecticide: 7; spread fire ash: 3; smear mud: 9; other: 10
		Yes	65 (82.3)	

**Table 3:** Characteristics of the participants: socioeconomic risk factors, domestic animals.

	Characteristics	*n* = 79	No. (%) Unless Specified	Details
Socioeconomic risk factors	Material of the roof of the house	Iron	79 (100.0)	
	Material of the walls in the house	Stone	1 (1.3)	
		Mud	77 (97.5)	
		Thatch	1 (1.3)	
	Income sources of the parents	Have a job	1 (1.3)	
		Work on their own farm	69 (89.6)	
		Fishing	1 (1.3)	
		Selling things in the market/village	6 (7.8)	
	No. of adults living in the household		median 2 [min, 1; max, 5]	
	No. of children living in the household		median 3 [min, 1; max, 12]	
	No. of people sleeping in the room with the patient		median 5 [min, 1; max, 8]	
	Sleeping place	Bed	33 (41.8)	
		Floor	46 (58.2)	
	Type of toilet at home	Ventilated pit latrine	26 (32.9)	
		Traditional latrine	52 (65.8)	
		Bush	1 (1.3)	
	Place to get water	Tap on the compound	26 (32.9)	
		Shared community tap	39 (49.4)	
		Community well	14 (17.7)	
	Time to get water (min)		mean 34.09	
	Assets owned by the family			Radio: 3; TV: 9; Mobile phone: 25; Bicycle: 3; Motorcycle: 4; Car: 0; Solar lamp: 11; None of these: 26
Domestic animals	Owns chickens	No	73 (92.4)	
		Yes	6 (7.6)	
	Owns pigs	No	71 (89.9)	
		Yes	8 (10.1)	

**Table 4:** Cure rates among the KMnO_4_ and 3% phenothrin lotion (single- and two-dose) treatment groups.

Treatment	KMnO_4_			3% Phenothrin Lotion Single Dose			3% Phenothrin lotion Two Doses		
	No.	%	95% CI	No.	%	95% CI	No.	%	95% CI
Day	213			129			73		
1	0			0			0		
4	2	0.9	0.1–3.4	15	11.6	6.7–18.5	0	n.a.	n.a.
7	25	11.7	6.8–16.3	28	21.7	14.9–29.8	6	8.2	3.1–17.0
10	94	44.1	37.0–50.1	58	45.0	36.2–53.9	31	42.5	31.0–54.6
14	148	69.5	63.3–75.7	92	71.3	62.7–78.9	49	67.1	55.1–77.7

KMnO_4_, potassium permanganate; CI, confidence interval.

**Table 5:** Absolute cure rate differences between KMnO_4_ and 3% phenothrin lotion (single and two doses).

Treatment	KMnO_4_ vs. 3% Phenothrin Lotion Single Dose			KMnO_4_ vs. 3% Phenothrin Lotion Two Doses			3% Phenothrin Lotion Single Dose vs. Two Doses		
	%	95% CI	*p*-Value *	%	95% CI	*p*-Value *	%	95% CI	*p*-Value *
Day									
4	10.7	5.0–16.4	<0.01	n.a.	n.a.	n.a.	n.a.	n.a.	n.a.
7	10.0	1.6–18.3	0.013	3.5	−4.1 to 11.1	0.37	13.5	3.9–23.0	0.005
10	0.8	−10.0 to 11.7	0.88	1.6	−11.5 to 14.8	0.80	2.5	−11.7 to 16.7	0.73
14	1.8	−8.1 to 11.8	0.72	2.4	−10.1 to 14.8	0.71	4.2	−9.1 to 17.5	0.57

*: by chi-square test. KMnO_4_, potassium permanganate; CI, confidence interval.

**Table 6:** Severity of itching in KMnO_4_ and 3% phenothrin lotion treatment groups (single and two dose groups).

Treatment	KMnO_4_			3% Phenothrin Lotion Single Dose			3% Phenothrin Lotion Two-Doses		
	No.	%	95% CI	No.	%	95% CI	No.	%	95% CI
Day	79			51			28		
1 Pre	13	16.5	9.1–26.5	11	21.6	11.3–35.3	2	7.14	0.9–23.5
1 Post	1	1.3	0.03–6.9	1	2.0	0.1–10.5	0	0	n.a.
4	8	10.1	4.5–19.0	7	13.7	5.7–26.3	0	0	n.a.
7	9	11.4	5.3–20.5	2	3.9	0.5–13.5	1	3.6	0.09–18.3
10	4	5.1	1.4–12.5	2	3.9	0.5–13.5	1	3.6	0.09–18.3
14	1	1.3	0.03–6.9	1	2.0	0.1–10.5	0	0.0	n.a.

KMnO_4_, potassium permanganate; CI, confidence interval.

**Table 7:** Severity of pain in KMnO_4_ and 3% phenothrin lotion treatment groups (single and two doses).

Treatment	KMnO_4_			3% Phenothrin Lotion Single Dose			3% Phenothrin Lotion Two Doses		
	No.	%	95% CI	No.	%	95% CI	No.	%	95% CI
Day	79			51			28		
1 Pre	3	3.8	0.8–10.7	2	3.9	0.5–13.5	1	3.6	0.09–18.3
1 Post	1	1.3	0.03–6.9	1	2.0	0.1–10.5	0	0	n.a.
4	5	6.3	2.1–14.2	4	7.8	2.2–18.9	0	0	n.a.
7	9	11.4	4.4–18.4	2	3.9	0.5–13.5	1	3.6	0.09–18.3
10	2	2.5	0.3–8.9	2	3.9	0.5–13.5	1	3.6	0.09–18.3
14	1	1.3	0.03–6.9	1	2.0	0.1–10.5	0	0.0	n.a.

KMnO_4_, potassium permanganate; CI, confidence interval.

## Data Availability

The data used during the analysis are available from the corresponding author upon reasonable request. The data are not publicly available due to privacy or ethical restrictions.

## Supplementary Materials

The following supporting information can be downloaded at: https://www.mdpi.com/article/10.3390/tropicalmed11020047/s1, Table S1: Number and proportion of dead fleas after treatment with absolute cure-rate difference and odds ratio; Table S2: Number and proportion of foot with itching by treatment; Table S3: Number and proportion of foot with pain by treatment.

## Abbreviations

The following abbreviations are used in this manuscript:

CHP	Community health promotor
EZR	Easy R
FTU	Finger-tip unit
KMnO_4_	Potassium permanganate
CU	Community unit
CI	Confidence interval
